# Prevalence and Prognostic Significance of Frailty in Gerontal Inpatients With Pre-clinical Heart Failure: A Subgroup Analysis of a Prospective Observational Cohort Study in China

**DOI:** 10.3389/fcvm.2020.607439

**Published:** 2020-12-10

**Authors:** Pei-Pei Zheng, Si-Min Yao, Jing Shi, Yu-Hao Wan, Di Guo, Ling-Ling Cui, Ning Sun, Hua Wang, Jie-Fu Yang

**Affiliations:** ^1^Peking University Fifth School of Clinical Medicine, Beijing, China; ^2^Department of Cardiology, Beijing Hospital, National Center of Gerontology, Institute of Geriatric Medicine, Chinese Academy of Medical Sciences, Beijing, China; ^3^Beijing Institute of Geriatrics, Beijing Hospital, National Center of Gerontology, National Health Commission, Institute of Geriatric Medicine, Chinese Academy of Medical Sciences, Beijing, China

**Keywords:** frailty, gerontal inpatients, NT-ProBNP, prognosis, pre-clinical heart failure

## Abstract

**Objective:** To evaluate the prognostic value of frailty in gerontal pre-clinical heart failure (stage B heart failure, SBHF) inpatients.

**Background:** The association between frailty and SBHF remains unknown.

**Methods:** We conducted a subgroup analysis of a prospective observational cohort study on frailty. The previous study recruited 1,000 elderly inpatients who were consecutively admitted to a tertiary referral hospital in Beijing, China, from September 2018 to February 2019. The outcomes were all-cause death or readmission at 1-year follow-up. SBHF was diagnosed for asymptomatic cardiac structural or functional abnormalities. Frailty was assessed using the Comprehensive Geriatric Assessment-Frailty Index (CGA-FI).

**Results:** Overall, 531 inpatients aged ≥65 years were deemed to have SBHF and followed up for 1 year. Of them, 34.5% exhibited frailty. During the follow-up period, all-cause death or readmission occurred in 157 (29.5%) participants. Of these participants, 36.6% (67/183) and 25.9% (90/348) belonged to the frail and non-frail groups, respectively (χ^2^ = 6.655, *P* = 0.010). Frailty, defined by the CGA-FI, rather than Fried frailty phenotype, could independently predict 1-year all-cause death or readmission (hazard ratio, 1.56; 95% confidence interval, 1.03–2.35; *P* = 0.034) and was more suitable for predicting all-cause death or readmission than N-terminal pro-B-type natriuretic peptide in female SBHF inpatients aged 80 years or over(AUC_CGA−FI_ vs. AUC_NT−proBNP_ 0.654 vs. 0.575, *P* = 0.017).

**Conclusions:** Frailty is highly prevalent even among SBHF inpatients aged ≥65 years. The CGA-FI can independently predict 1-year all-cause death or readmission, rather than Fried frailty phenotype. Frailty in gerontal SBHF inpatients deserves more attention.

**Clinical Trial registration:** ChiCTR1800017204; date of registration: 07/18/2018.

## Introduction

Heart failure (HF) is a complex clinical syndrome caused by any structural or functional impairment in the ventricle's ability to fill with or eject blood (1). The prevalence of HF increases with age and is more than 10% in individuals over 70 years of age (2). HF is an important cause of hospitalization in elderly patients (3), and the 5-year mortality after hospitalization for decompensated HF is >75% (4).

Frailty is the most problematic expression of population aging and is considered a geriatric syndrome of diminished reserve and resistance to stressors due to cumulative declines across different physiological systems, with features of weakness, reduced endurance, and slowed performance (5, 6). Approximately 10–20% of adults aged >65 years exhibit frailty, and the prevalence doubles in those aged >85 years; furthermore, frail old adults are at a higher risk for adverse health outcomes (7). Frailty is particularly important in HF as it places gerontal patients in repeated situations of stress and vulnerability and promotes frailty at a prevalence of nearly 50% (8). Similarly, the incidence of HF increases by 30% in frail patients. Frailty increases all-cause mortality, hospitalization, disability, drug adverse reactions, and social support for HF (9, 10).

Therefore, HF and frailty are closely related to poor outcomes, especially when they coexist. Fortunately, both HF and frailty can be intervened to improve health status (11, 12). Early identification of frailty in HF patients, particularly HF patients at an earlier stage, is especially important. However, previous studies have mainly focused on frailty and acute HF (13), chronic HF (14), and HF with reduced or preserved ejection fraction (13)—that is, frailty and stage C/D HF rather than stage A/B HF. The prevalence of pre-clinical HF (stage B HF, SBHF) is ~30–44% among patients aged >65 years, which is almost twice that of stage C/D HF (12, 15). If the prevalence and prognostic value of frailty are increased in SBHF, assessment of frailty status in SBHF patients becomes essential for the early detection and prevention of adverse outcomes.

The prevalence and prognostic value of frailty in SBHF patients has not explored in China or other countries. Accordingly, we conducted a Frailty–SBHF Study to estimate the prevalence of frailty in SBHF inpatients aged ≥65 years and to evaluate the predictive value of frailty in SBHF inpatients.

## Methods

### Design

The Frailty–SBHF Study was a subgroup analysis of a prospective observational cohort study on frailty in China (16). Previously acquired data on demographics and clinical characteristics were used to assess interactions and relationships with the outcome.

### Participants

**Figure 1:**
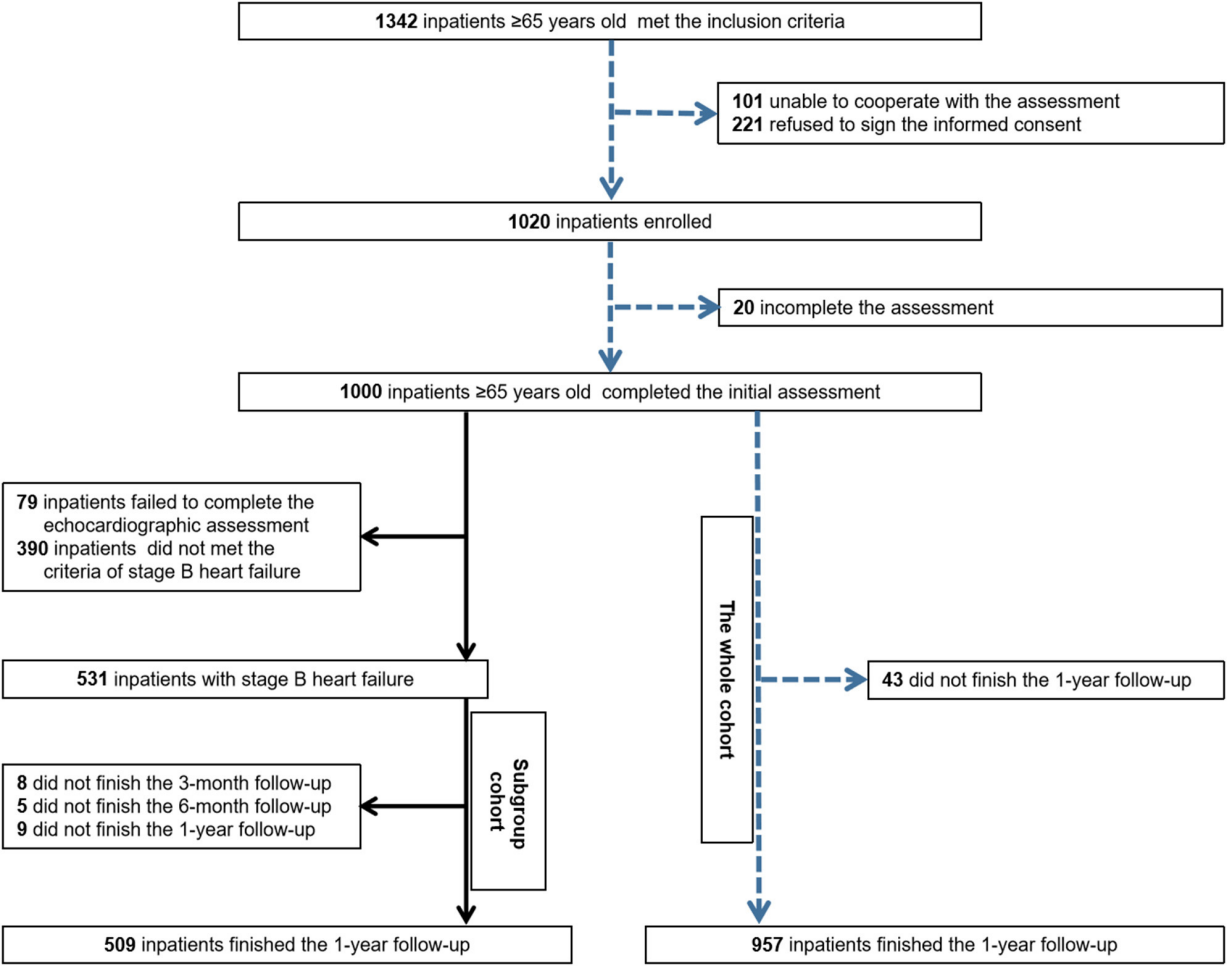
Flow chart of the whole prospective observational cohort and pre-clinical heart failure subgroup cohort.

The previous cohort study recruited 1,000 elderly inpatients who were consecutively admitted to a tertiary referral hospital in Beijing, China, from September 2018 to February 2019. The inclusion criterion was an age of 65 years or older, whereas the exclusion criteria were inability to cooperate with the assessment procedure and refusal to sign the informed consent form (16). All of them were screened to identify individuals with SBHF. Of these, 79 who failed to complete the echocardiographic assessment and 390 who did not meet the criteria for SBHF were excluded. Finally, 531 inpatients were enrolled in the Frailty–SBHF Study and followed up for 1 year (Figure 1). The trial was conducted in accordance with the ethical principles outlined in the Declaration of Helsinki, and the research protocol was approved by the Ethics Committee of Beijing Hospital (approval no. 2018BJYYEC-121-02). Written informed consent was obtained from the patients or their legal representatives. Information was gleaned by fixed investigators, who had passed the survey training test, through a case report form to ensure the validity of the collected data. Data were managed through Research Electronic Data Capture (REDCap) and the entire study was supervised by Peking University Clinical Research Institute.

All participants underwent comprehensive geriatric assessment, and their baseline data were collected, including age, sex, medical insurance, residence, job, whether they were living alone, years of education, smoking habit, drinking habit, HF risk factors, reasons for SBHF, atrial fibrillation or atrial flutter (AF/AFL), peripheral arterial disease, stroke (hemorrhagic stroke, ischemic stroke with activity capacity, lacunar infarction without symptom and transient ischemic attack), renal failure, estimated glomerular filtration rate, N-terminal pro-B-type natriuretic peptide (NT-proBNP) level, and echocardiographic data.

SBHF refers to asymptomatic cardiac structural or functional abnormalities that is strongly associated with HF development according to the 2013 ACC/AHA Guidelines for the Evaluation and Management of Chronic Heart Failure in the Adult (1). It includes left ventricular (LV) enlargement, LV hypertrophy, reduced LV ejection fraction (LVEF), diastolic dysfunction (DD), prior myocardial infarction, wall motion abnormalities, and valvular heart disease (12). Cardiac structure and function change with age, including smaller LV size, higher LVEF, and lower early diastolic mitral annular velocity (e′), as measured by tissue Doppler imaging. Considering the age and race of the population studied, our study combined these two age-specific criteria to define SBHF. The criteria by the Atherosclerosis Risk in Communities (ARIC) study defined 95% percentile limits derived from a healthy subgroup aged 67–91 years (17) and is widely used (15) The criteria by the Doppler Echocardiographic Measurements in Normal Chinese Adults (EMINCA) study defined 95% percentile limits derived from a healthy yellow-race subgroup aged 65 years or older (18). One will be diagnosed SBHF with at least one of the following:

1. valvular heart disease: moderate or greater stenosis or regurgitation in the aortic or mitral valve;
2. LV enlargement: LV end-diastolic volume (LVEDV)/body surface area (BSA) >60.2 mL/m^2^ (men) or >51.9 mL/m^2^ (women);
3. LV hypertrophy: LV mass/height^2.7^ >45 g/m^2.7^ (men) or >41.5 g/m^2.7^ (women);
4. DD: septal e′ <4.3 cm/s (men) or <4.1 cm/s (women); septal E/e′ ratio >14.8 (men) or >17.4 (women);left atrial anteroposterior diameter (LAAPD) >39.2 mm (men aged 65–69 years), >40.3 mm (men aged ≥70 years), >38.3 mm (women aged 65–69 years), or >38.6 mm (women aged ≥70 years);
5. Reduced LVEF: LVEF <54.6% (men aged 65–69 years), <53% (men aged ≥70 years), <54.5% (women aged 65–69 years), or <53.5% (women aged ≥70 years).

Frailty was evaluated using the Comprehensive Geriatric Assessment-Frailty Index (CGA-FI) proposed by Rockwood and based on which the suitable FI could be created according to the characteristics of different populations (5). In our previous study, 48 variables were selected to construct the CGA-FI, including activities of daily living, chronic diseases, depression, anxiety, loneliness, Mini-Mental State Examination, geriatric syndrome, insomnia, body mass index, calf circumference, peak flow, grip strength, and 4-m walking speed. A CGA-FI score of 0.25 or more indicated frailty, and the CGA-FI proved to be an optimal tool for frailty assessment in the participants of our cohort (16).

The composite endpoint was 1-year all-cause death or readmission. We confirmed the event occurrence through telephone interviews with the participant or caregiver at 1 year after the date of initial admission and through medical record review, if necessary.

### Statistical Analysis

To maximize statistical power and minimize bias that may occur if inpatients with missing data were excluded from analyses, multiple imputation by chained equations was used for missing values in the covariates of adjusted statistical models, and five complete data sets were obtained.

Shapiro–Wilk tests and quantile-quantile plots were used to evaluate continuous variables for normal distribution. To describe baseline characteristics, percentages, mean ± standard deviation, and the median [interquartile range: 25th to 75th percentiles] were used for categorical variables, normally distributed continuous variables, and non-normally distributed continuous data, respectively. The Pearson χ^2^ test, two-sample *t*-test, and Mann–Whitney *U*-test were employed, as appropriate, to detect any differences in baseline study variables between groups (non-frail vs. frail).

Survival without death or readmission was calculated in days and estimated using the Kaplan–Meier method. The log-rank test was used to compare the two groups. Multivariate Cox proportional hazards models were utilized to describe the association between frailty and 1-year all-cause death or readmission after adjustment for age, sex, and NT-proBNP level, which were selected according to clinical relevance. The best NT-proBNP cut-off point for death or readmission was explored by survival classification and regression tree (CART) analysis. The proportional hazards assumption for the Cox model was checked using Schoenfeld residuals, and no violation was found. The results of Cox proportional hazards regression model were expressed as hazard ratios (HR) and 95% confidence intervals (CIs). We also used receiver operating characteristic C-statistics to evaluate the level of prognostic value of CGA-FI, NT-proBNP, and NT-proBNP + CGA-FI (FN) in all participants and participants aged ≥80 years. FN was defined by including high NT-proBNP level as one component to the CGA-FI (high NT-proBNP level was determined by CART analysis).

Sensitivity analysis was conducted as follows: [1] analysis with frailty evaluated by the Fried frailty phenotype [defined by Fried et al.'s Cardiovascular Health Study (6)] to achieve the maximum statistical power of the prognostic value of frailty and [2] analysis of the prognostic value of CGA-FI, NT-proBNP, and NT-proBNP+CGA-FI according to the participants' sex to assess whether the results of primary analysis were driven by sex.

All statistical tests were two-tailed, and a *P* < 0.05 was considered statistically significant. SPSS software version 25 (IBM Corp., Armonk, NY, USA) was used to perform statistical analysis. GraphPad Prism version 6.01 (GraphPad Software Inc., San Diego, CA, USA) was used for figure generation.

## Results

### Baseline Characteristics

Participants' mean age was 75.5 years (range: 65–92.7), and 228 (42.9%) participants were male. A total of 348 participants (65.5%) comprised the non-frailty group, whereas 183 participants (34.5%) comprised the frailty group. Table 1 showed various baseline characteristics. Frail gerontal SBHF patients were older and living alone, had fewer years of education, a higher level of CCI and NT-proBNP, a smaller proportion of drinking, and larger proportion of hypertension, diabetes, peripheral arterial disease, stroke, and renal failure. The type of SBHF was more often DD and less often LV enlargement in frail SBHF inpatients.

**Table 1:** Baseline and heart failure characteristics of all participants.

	**Overall *n* = 531**	**Non-frail *n* = 348 (65.5%)**	**Frail *n* = 183 (34.5%)**	***P*-value (pooled)**
**Demographics**				
Age, years	75.5 ± 6.48	73.7 ± 5.81	78.9 ± 6.35	<0.001
Male sex	228 (42.9)	159 (45.7)	69 (37.7)	0.077
Medical insurance	528 (99.4)	345 (99.1)	183 (100)	0.208
Residence, city	511 (96.2)	332 (95.4)	179 (97.8)	0.165
Manual worker	197 (37.1)	124 (35.6)	73 (39.9)	0.334
Living alone	48 (9.0)	25 (7.2)	23 (12.6)	0.040
Education, years	10.7 ± 4.30	11.1 ± 4.22	9.94 ± 4.36	0.004
Current smoker	42 (7.9)	24 (6.9)	18 (9.8)	0.483
Current drinker	104 (20.2)	83 (23.9)	24 (13.1)	0.013
**Risk factors of HF**				
Hypertension	409 (77.0)	254 (73.0)	155 (84.7)	0.002
Diabetes	178 (33.5)	106 (30.5)	72 (39.3)	0.039
Obesity	119 (22.4)	74 (21.3)	45 (24.6)	0.382
Coronary artery disease	291 (54.8)	197 (56.6)	94 (51.4)	0.249
**Reasons for pre-clinical HF**				
LV hypertrophy	270 (51.5)	174 (51.0)	96 (52.5)	0.754
LV enlargement	319 (61.0)	218 (64.1)	101 (55.2)	0.046
Reduced LVEF	18 (3.4)	12 (3.5)	6 (3.3)	0.890
Diastolic dysfunction	276 (52.7)	167 (49.0)	109 (59.6)	0.021
Myocardial infarction (WMAs)	66 (12.4)	49 (14.1)	17 (9.3)	0.112
Valvular heart disease	35 (6.7)	23 (6.7)	12 (6.6)	0.935
**Other comorbidities**				
Atrial fibrillation or atrial flutter	77 (14.5)	43 (12.4)	34 (18.6)	0.053
Peripheral arterial disease	88 (16.6)	39 (11.2)	49 (26.8)	<0.001
Stroke	124 (23.4)	53 (15.2)	71 (38.8)	<0.001
Renal failure	77 (14.5)	41 (11.8)	36 (19.7)	0.014
**Laboratory test results**				
eGFR <60 ml/min*1.73^2^	67 (12.6)	34 (9.8)	33 (18.0)	0.006
NT-proBNP level, pg/ml	162 [79.5, 364]	144 [64.7, 294]	231 [117, 505]	<0.001

*Values are shown as mean ± standard deviation, median [interquartile range: 25th to 75th percentiles], or n (%). Data were analyzed using the t-test for normally distributed continuous data, Mann-Whitney U-test for non-normally distributed continuous data, and χ^2^ test for categorical data. HF, heart failure; LV, left ventricular; LVEF, left ventricular ejection fraction; WAMs, wall-motion abnormalities; eGFR, estimated glomerular filtration rate*.

Measures of the left atrium, right ventricle, left ventricle, LV function, and valve abnormality are presented in Table 1. Frail participants with SBHF showed larger LAAPD and septal E/e′ ratio, as well as reduced LVEDV/BSA and lower septal e′.

### Frailty and 1-Year Death or Readmission

**Figure 2:**
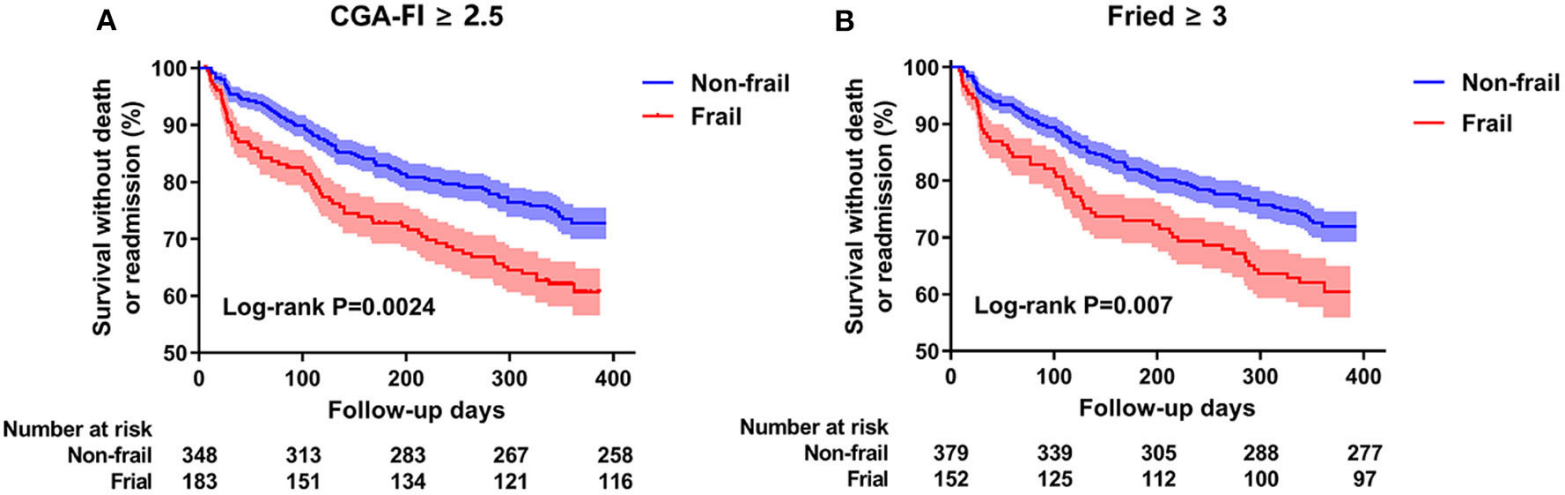
Kaplan-Meier survival curves by frailty in all participants. **(A)** stands for the frailty defined by CGA-Fl. **(B)** represents the frailty defined by Fried frailty phenotype. Event rates of 1-year death or readmission have been analyzed by a log-rank test.

During the follow-up period, all-cause death or readmission occurred in 157 (29.5%) participants (8 deaths and 153 readmissions); of these participants, 36.6% (67/183) and 25.9% (90/348) belonged to the frail and non-frail groups, respectively (χ^2^ = 6.655, *P* = 0.010). Besides, there was only 2.7% (5/183) in frail and 0.9% (3/348) in non-frail group death or readmission for heart failure manifestation (stage C). The mean survival times without death or readmission were 286 ± 10.7 days for the frail participants and 328 ± 6.5 days for the non-frail participants (Figure 2A). The sensitivity analysis with frailty evaluated by the Fried frailty phenotype showed the same trend (Figure 2B).

**Figure 3:**
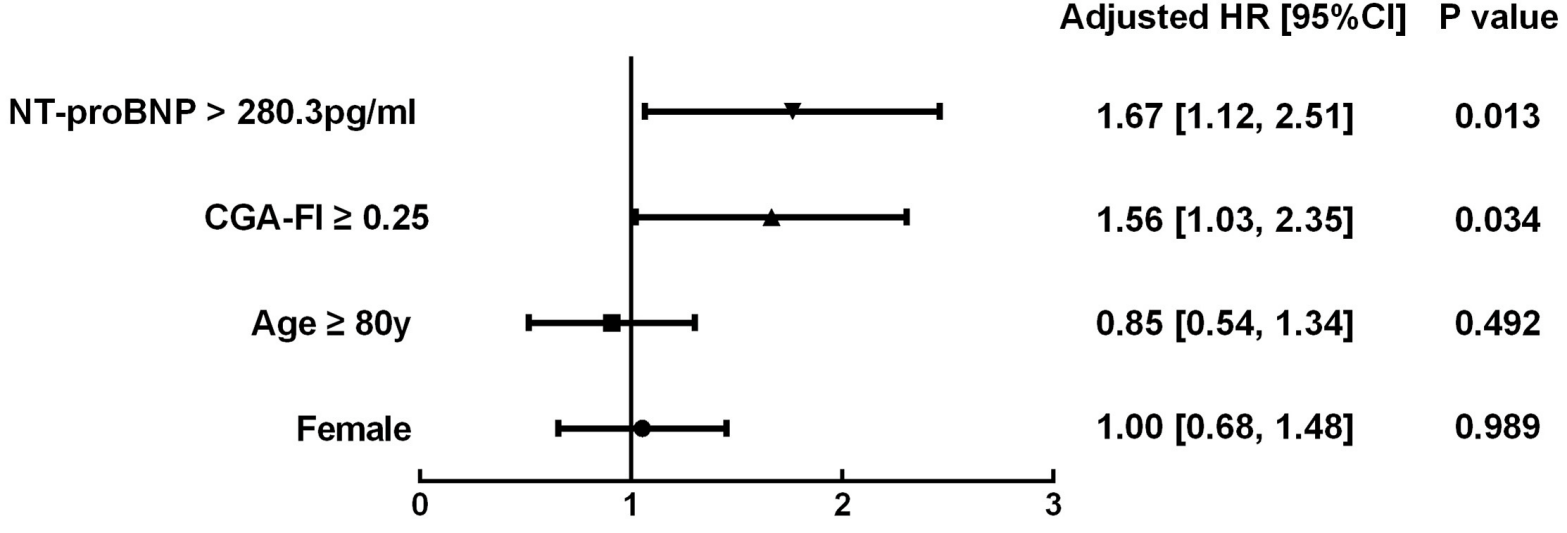
Multivariable Cox proportional hazard models of frailty in all participants, controlling for potential confounding factors (gender, age and NT-proBNP). Frailty was defined by CGA-Fl ≥ 0.25 and events were defined as 1-year all-cause death or readmission. HR, Hazard ratio; CI, confidence intervals.

**Figure 4:**
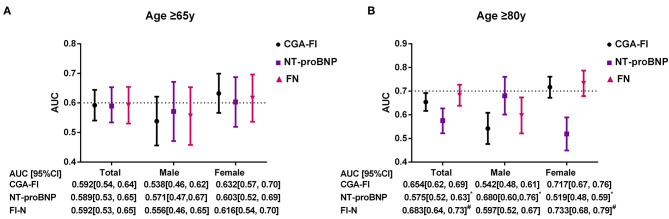
C-statistics for the prediction of 1-year death and readmission by CGA-FI, NT-proBNP, and FN, estimated from time-dependent ROC curves in all, male and female participants [**(A)** for all participants; **(B)** for participants aged 80 years or older]. ROC, receiver operating characteristic; AUC, area under curve; FN, NT-proBNP added CGA-Fl; CI, confidence interval. *P < 0.05, compared with CGA-Fl; ^#^*P* < 0.05, compared with NT-proBNP.

The survival CART analysis revealed the NT-proBNP cut-off point for predicting death or readmission in participants with SBHF and we showed that patients with an NT-proBNP level of ≥280.3 pg/mL experienced a high incidence of death or readmission (Figure 3, HR, 1.67; 95% CI, 1.12–2.51; *P* = 0.013). More importantly, frailty was associated with a 1.56-fold increase in 1-year death or readmission risk (95% CI, 1.03–2.35; *P* = 0.034) after adjustment for age, sex, and NT-proBNP level. However, frailty defined by the Fried frailty phenotype could not independently predict death or readmission (*P* > 0.05). Finally, we included an NT-proBNP level of ≥280.3 pg/mL as an additional parameter to CGA-FI (FN) and compared its predictive value with that of CGA-FI and NT-proBNP (Figure 4). There was no significant difference between each of them in all participants (Figure 4A). Nevertheless, in participants aged ≥80 years, the predictive value of CGA-FI was higher than that of NT-proBNP (Figure 4B, AUC_CGA−FI_ vs. AUC_NT−proBNP_ 0.654 vs. 0.575, *P* = 0.017; AUC_FN_ vs. AUC_NT−proBNP_ 0.683 vs. 0.575, *P* = 0.002), especially in women.

## Discussion

The present study is the first study to investigate frailty, as defined by the CGA-FI, in gerontal SBHF inpatients to clarify our understanding about the burden of frailty at an early stage of HF. It demonstrates the importance of frailty as a biological syndrome, which is not included in most prognostic models of HF.

### Frailty Prevalence

The prevalence rate of frailty in SBHF inpatients is similar to or lower than that for elderly participants with clinical HF using the FI criteria (35–65%) (14, 19). Obviously, the rate is much higher than those for patients without SBHF or stage C/D HF in our cohort (27.7%, which has not been published) and community-dwelling elderly participants without clinical HF [3% in the group aged 65–70 years using the Fried frailty phenotype criteria (6, 10); 7% in the group aged ≥65 years using the deficit index (14)]. Frailty and HF share common mechanistic features, including strong relationships with a high burden of comorbidities, aging, and inflammation (10). Furthermore, individuals with SBHF have more comorbidities than those without HF (20). The above-mentioned mechanistic features will be strengthened once HF starts. This may partly elucidate the higher prevalence of SBHF in elderly inpatients than in the general population.

### Frailty Predicts 1-Year All-Cause Death or Readmission

Frailty conferred an independently increasing risk of all-cause death or readmission during the 1st year after discharge in gerontal SBHF inpatients. This finding is coincident with that of other studies that report the predictive value of frailty in the general population (6) and clinical HF patients (21). The NT-proBNP level increases with the HF stage (22) and predicts mortality and readmission among patients aged ≥65 years who are hospitalized for HF (23). Our study also showed its predictive value for 1-year death or readmission in gerontal SBHF inpatients. However, its predictive value was lower than that of frailty in female inpatients aged ≥80 years. We suggest possible reasons for this discrepancy. First, women had a higher rate of frailty than men (37.6% vs. 30.3%, χ^2^ = 3.121, *P* = 0.047), and frailty increases the risk of readmission independently (10). Second, anxiety and depression disorders contribute to a higher readmission and are more common among women than among men, and elderly female patients have worse drug sensitivity and treatment responses to medications (24). Inclusion of these components in the CGA-FI may have led to a higher predictive value, as compared to NT-proBNP. Third, we found that women had a lower NT-proBNP level than men (324 vs. 368, *t* = 2.012, *P* = 0.036), which is consistent with a previous study (25), leading to the poor sensitivity of NT-proBNP in women. More efforts are needed to explore this difference. The independent predictive value of frailty indicates the importance of frailty assessment and intervention.

### Frailty Assessment Tool

Various tools for frailty evaluation exist, all of which are mainly based on physical frailty and multidimensional frailty. We found that Fried frailty phenotype was a factor but not an independent factor for predicting 1-year death in gerontal SBHF inpatients because of its low sensitivity such that some important frailty types were not recognized. In comparison, the CGA-FI used in our study combines two concepts and is not only a better predictor but also a diagnostic method with the highest sensitivity (94.8%) and good specificity (87.0%) for the frailty identification in our cohort (16).

### Frailty and Other Concerns

On comparing the echocardiographic data between frail and non-frail participants, DD was closely related to frailty in SBHF inpatients, as mainly manifested by a larger LAAPD and septal E/e′ ratio, as well as lower septal e′. Considering the higher prevalence of hypertension and slightly increased LV mass in frailty, we think that frail individuals were more often presented a DD status similar to the concentric LV geometry caused by hypertension (26). Furthermore, we found a lower proportion of LV enlargement in frailty. In fact, LVEDV became smaller with increasing age, and older age may account for it in frail individuals (17). Nonetheless, no similar study has investigated this phenomenon; hence, further studies need to be conducted.

## Limitations

Our study has several limitations. Firstly, our study participants were from a single tertiary hospital, which may have influenced the generalizability of our results. Secondly, there is no acknowledged and suitable criterion to recognize structural heart disease in elderly Chinese patients thus far. Thirdly, the current study did not have a sufficiently long follow-up, so as to the incidence of death or readmission for heart failure manifestation was quite low. Multicenter or community studies with a larger sample size, longer follow-up, and specific heart disease related endpoints are warranted in the future.

## Conclusion

Frailty is highly prevalent even among SBHF inpatients aged ≥65 years. CGA-FI is superior to Fried frailty phenotype in independently predicting 1-year all-cause death or readmission. Both frailty and SBHF are partly reversible conditions and deserve more attention for the early detection of and improvement in adverse outcomes.

## Data Availability Statement

The raw data supporting the conclusions of this article will be made available by the authors, without undue reservation.

## Ethics Statement

The studies involving human participants were reviewed and approved by Ethics Committee of Beijing Hospital. The patients/participants provided their written informed consent to participate in this study.

## Author Contributions

HW and J-FY provided the conception of the idea for the study. P-PZ and S-MY contributed to the development of the methodology and wrote the manuscript. JS analyzed the acquired data. Y-HW, DG, L-LC, and NS were responsible for the interpretation of statistical results. HW revised the manuscript. All authors contributed to the article and approved the submitted version.

## Conflict of Interest

The authors declare that the research was conducted in the absence of any commercial or financial relationships that could be construed as a potential conflict of interest.
